# A Systematic Review of Vascular Structure and Function in Pre-eclampsia: Non-invasive Assessment and Mechanistic Links

**DOI:** 10.3389/fcvm.2019.00166

**Published:** 2019-11-15

**Authors:** Shady Kirollos, Michael Skilton, Sanjay Patel, Clare Arnott

**Affiliations:** ^1^Sydney Medical School, University of Sydney, Sydney, NSW, Australia; ^2^Boden Collaboration for Obesity, Nutrition, Exercise, and Eating Disorders, Faculty of Medicine and Health University of Sydney, Sydney, NSW, Australia; ^3^Department of Cardiology, Royal Prince Alfred Hospital, Sydney, NSW, Australia; ^4^Department of Coronary Diseases, The Heart Research Institute, Sydney, NSW, Australia; ^5^Department of Cardiology, The George Institute for Global Health, Sydney, NSW, Australia

**Keywords:** pre-eclampsia, vascular changes, endothelial dysfunction, cardiovascular disease, pregnancy

## Abstract

Hypertensive disorders of pregnancy, such as pre-eclampsia, are known to be independently associated with the development of premature cardiovascular disease (CVD) in women. In pre-eclampsia, the placenta secretes excess anti-angiogenic factors into the maternal circulation, leading to widespread endothelial damage, and inflammation. This endothelial damage is evidenced to persist beyond the acute illness. However, whether it is permanent and responsible for the elevated rates of premature CVD seen in this at-risk group remains unclear. A systematic review of the available literature with respect to vascular structure and function prior to, during and after a pregnancy complicated by pre-eclampsia was performed. Studies non-invasively assessing vascular structure using carotid intima-media thickness (CIMT), retinal microvasculature caliber, CT coronary angiogram, or coronary calcium scores were included. Vascular function was assessed using brachial flow-mediated dilation (FMD), pulse wave analysis (PWA), and peripheral arterial tonometry (PAT). In total 59 articles were included (13 CIMT, 5 CTCA/Ca score, five retinal microvasculature, 27 FMD, 7 PAT, and 14 PWV/PWA), consisting of prospective and retrospective cohort, and case-control studies. Change in vascular structure was evidenced with significant increases in CIMT by 73–180 μm greater than that of non-affected women. This is tempered by other studies reporting resolution of structural changes postpartum, highlighting the need for further research. Accelerated coronary calcification and plaque deposition was identified, with greater rates of increased calcium scores and subclinical coronary artery disease shown by CTCA in women with a history of pre-eclampsia at 30 years postpartum. Impaired endothelial function was consistently reported prior to, during and immediately after pregnancy as evidenced by differences in FMD of 1.7–12.2% less than non-affected women, an increase in PWV by 13.2–26%, and reduced retinal microvascular caliber and arterial elasticity indices. The evidence was less conclusive for the persistence of long-term endothelial dysfunction. Understanding the underlying mechanistic links between pre-eclampsia and CVD is a key step to identifying targeted therapies aimed at “repairing the endothelium” and attenuating risk. This review has highlighted the need for a greater understanding of vascular structure and function following pre-eclampsia through high quality studies with large sample sizes, particularly in the longer postpartum period when clinical CVD disease starts to manifest.

## Introduction

Pre-eclampsia (PE) is a malignant syndrome of pregnancy mediated by abnormal placentation and subsequent dysfunction. It affects 2–8% of all pregnancies (1) and occurs most commonly in the third trimester but may also develop intrapartum or early postpartum (2). Pre-eclampsia is characterized by hypertension (>140/90 mmHg) after 20 weeks gestation, with either proteinuria, maternal organ dysfunction (hematological, renal, neurological or hepatic), or uteroplacental dysfunction such as fetal growth restriction (2). Globally, it is a leading cause of both maternal and fetal death (3).

The relevance of PE to maternal health, however, extends beyond the acute pregnancy and early postpartum period. PE has been identified in numerous studies to be an independent risk factor for the development of premature maternal cardiovascular disease (CVD). These studies include cohort and case-control studies of both retrospective and prospective design; and several systematic reviews and meta-analysis of large populations (up to 2 million women). Each study population varies in terms of patient composition, severity of the illness, maternal age and follow-up period. Although this heterogeneity naturally leads to variations in specific disease hazard ratios, they all demonstrate a strong association between CVD and PE. The CHAMPS study, for example, demonstrated a more than doubling of the risk of CVD in women who had experienced a pre-eclamptic pregnancy as compared to women with an unaffected pregnancy (HR 2.1; 95% CI, 1.8–2.4) (4).

Whilst the epidemiological link has been clearly demonstrated, the mechanisms through which PE potentially confers this elevated risk of CVD are incompletely understood, nor is it proven to be a causal relationship. Pathological failure in the vascular remodeling of maternal spiral arteries and resultant hypoperfusion of the placenta is integral to the development of PE (1). This sets off an acute cascade of oxidative stress, inflammation and endothelial dysfunction. Systemic endothelial dysfunction has also been demonstrated to be involved in the development and progression of atherosclerosis and kidney disease, and thus it has been postulated that this may explain the link between PE and CVD (5). Whether pre-eclampsia-induced inflammation and endothelial function persist beyond pregnancy, however, has not been conclusively elucidated.

A further challenge in the understanding of PE and its vascular ramifications is that rather than being one single disease process, it is likely comprised of distinct phenotypes with differing long-term risk profiles. Early-onset PE (onset before 34 weeks gestation) has been linked to impaired trophoblastic differentiation within maternal spiral arteries causing placental hypoxia, release of placental debris such as sFlt1 (soluble fms-like tyrosine kinase) and soluble endoglin (sEng) into maternal circulation and thereby systemic inflammation and oxidative stress (6). Whereas, late-onset PE (>34 weeks at disease onset) is more often associated with pre-existing maternal conditions/cardiometabolic risk factors that may compromise the integrity of the endothelium (6). Current evidence suggests that early-onset PE is a stronger risk factor for the development of CVD than the later onset form (7).

To date, there has been no comprehensive review of the available evidence with respect to maternal vascular structure and function in association with a pre-eclamptic pregnancy. The aim of this systematic review, therefore, is to evaluate the current evidence base with respect to vascular structure and function prior to disease onset, during PE, immediately postpartum and long term. This will involve analysis via non-invasive modalities including flow mediated dilation (FMD), peripheral arterial tonometry (PAT), carotid intima-media thickness (CIMT), retinal microvasculature, pulse wave analysis and velocity (PWA/PWV), CT coronary angiogram, and calcium scores. The evidence presented intends to shed light on the pathophysiological links between PE and CVD.

### Method

We performed a systematic review of studies reporting on non-invasive assessment of endothelial structure and function in association with PE (prior, during, after). This review was conducted in accordance with the Preferred Reporting Items of Systematic Reviews and Meta-Analyses (PRISMA) statement (8).

### Search Strategy, Study Selection, and Data Extraction

Medline via Ovid (from 1946 to September 2019) and EMBASE via Ovid (from 1980 to September 2019) were searched systematically for relevant trials (Table 1). The search had no language restriction and used subject headings relevant to PE and hypertensive disorders of pregnancy.

**Table 1:** Electronic database search terms.

***MEDLINE via OVID***	***EMBASE via OVID***
1 Pre-eclampsia.mp. or Pre-Eclampsia/ 2 Hypertension, Pregnancy-Induced/, or Pre-Eclampsia/ or Pregnancy complications, Cardiovascular/ or Hypertensive disorder of pregnancy.mp. 3 Gestational Hypertension.mp. or Hypertension, Pregnancy-Induced/ 4 Intima media thickness.mp. Carotid Intima-Media Thickness/ 5 Retinal Microvasculature.mp. 6 Flow mediated dilatation.mp. 7 Pulse wave velocity.mp. or Pulse Wave Analysis/ 8 Computed Tomography Angiography/ or Tomography, X-Ray Computed/ or Coronary Angiography/ or CT coronary angiography.mp. 9 Peripheral arterial tonometry.mp. 10 Vascular structure.mp. 11 Endothelial dysfunction.mp. 12 Endothelium, Vascular/ or endothelial function.mp. 13 vascular function.mp. 14 1 or 2 or 3 15 4 or 5 or 6 or 7 or 8 or 9 16 10 or 11 or 12 or 13 17 humans.mp. or Humans/ 18 14 and 15 and 16 and 17	1 Pre-eclampsia.mp. or Pre-Eclampsia/ 2 Hypertension, Pregnancy-Induced/ or Pre-Eclampsia/ or Pregnancy complications, Cardiovascular/ or Hypertensive disorder of pregnancy.mp. 3 Gestational Hypertension.mp. or Hypertension, Pregnancy-Induced/ 4 Intima media thickness.mp. Carotid Intima-Media Thickness/ 5 Retinal Microvasculature.mp. 6 Flow mediated dilatation.mp. 7 Pulse wave velocity.mp. or Pulse Wave Analysis/ 8 Computed Tomography Angiography/ or Tomography, X-Ray Computed/ or Coronary Angiography/ or CT coronary angiography.mp. 9 Peripheral arterial tonometry.mp. 10 Vascular structure.mp. 11 Endothelial dysfunction.mp. 12 Endothelium, Vascular/ or endothelial function.mp. 13 vascular function.mp. 14 1 or 2 or 3 15 4 or 5 or 6 or 7 or 8 or 9 16 10 or 11 or 12 or 13 17 humans.mp. or Humans/ 18 14 and 15 and 16 and 17

Our primary aim was to assess the vascular structure and function associated with PE using these key non-invasive modalities: Carotid intima media thickness, coronary artery calcification, retinal microvasculature, flow-mediated dilatation, peripheral arterial tonometry, and pulse wave analysis/velocity. The titles and abstracts of all identified articles were extracted and screened for an initial assessment of eligibility. Full text versions of potentially eligible studies were reviewed to reach a final decision on inclusion or exclusion. We excluded studies not conducted in humans, reviews, editorials, letters, non-English, abstract-only, and duplicate reports. Data were extracted into an electronic spreadsheet and review of trials for eligibility, data extraction, and quality assessment were conducted independently by two authors (SK, SP) using a standardized approach. Any disagreement was settled by consultation with a third author (CA).

The key outcomes studied were vascular structure and function, arterial stiffness and endothelial dysfunction. Reference lists of journal articles were also screened for additional citations that could be included in the search criteria. Given the heterogeneity of study design, cohorts and timing of investigations, a meta-analysis was not performed. The key results of the included studies were discussed methodically by investigative tool and timeline in the results.

## Results

### Systematic Review

**Figure 1:**
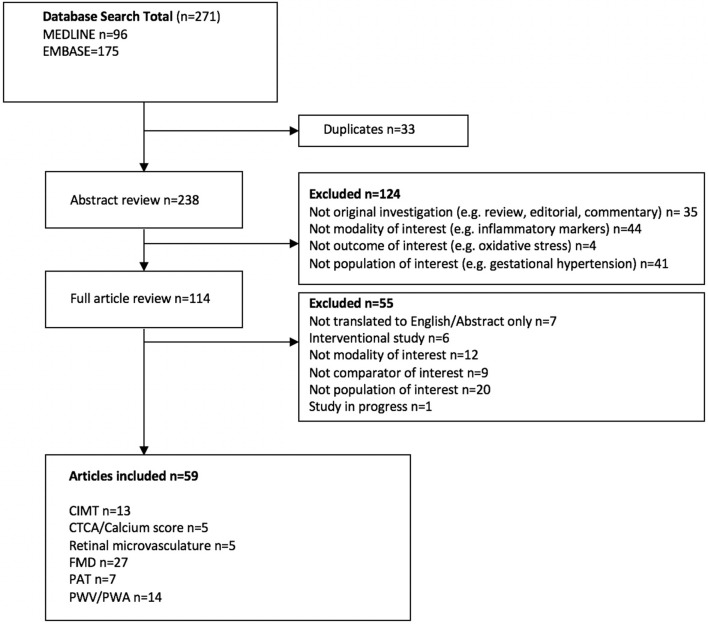
Flow diagram illustrating the process of study identification.

In total 59 studies were identified for inclusion; 23 related to vascular structure (13 CIMT, five CTCA or Ca score, five retinal microvasculature) and 48 relative to vascular function (27 FMD, 7 PAT, 14 PWV/PWA) were included, with several studies implementing more than one modality (Figure 1). A meta-analysis was not performed due to the heterogeneity of studies with respect to participant population, disease severity, multiple modality use, methodology and timing of follow-up. However, the key findings for each modality are described in detail below.

### Vascular Structure

#### Carotid Intima-Media Thickness

Carotid intima-media thickness (CIMT) is a well validated, non-invasive marker of pre-clinical atherosclerotic disease. It involves ultrasound evaluation of the thickness of the intimal and medial carotid arterial wall. Data have shown that an increased CIMT confers an elevated risk of coronary atherosclerotic lesions and future cardiovascular disease in both histological and epidemiological studies (9, 10).

CIMT is an attractive tool for the assessment of vascular structure in pregnancy as it is non-invasive and does not rely on the use of ionizing radiation. The key consistent findings from the systematic review of 13 available CIMT studies in PE is that CIMT increases during a pre-eclamptic pregnancy as compared to a non-effected pregnancy and persists up to 10 years postpartum.

Evidence supporting an increase in CIMT prior to PE onset, however, is limited. One study measuring CIMT in 601 pregnant women found that CIMT was significantly increased within the first trimester in the 143 who subsequently developed PE compared to the 420 women who remained normotensive (580 ± 100 vs. 340 ± 160 μm; *P* < 0.03) (11). Further to this, the women who subsequently develop gestational hypertension did not have a significantly increased CIMT.

There are several studies evaluating CIMT during pregnancy with varied cohorts and heterogenous results (12–14). For example, a prospective study of 22 women with late onset PE reported a significant increase of 108 μm in CIMT during pregnancy as compared to pregnant controls (459 ± 95 vs. 351 ± 85 μm, *P* = 0.0001) (15). Another study, comparing 50 early-onset and 50 late-onset PE cases determined that CIMT was significantly increased in late-onset PE compared to the normotensive controls, yet this increase was not significant in the early-onset group, even though similar blood pressures were observed between the two PE groups; this suggests distinct aetiological vascular changes contributing to the two forms of PE (13). Conversely, other studies have only identified elevated CIMT in women with chronic hypertension in pregnancy rather than PE (16). Whilst inconclusive, these studies raise the possibility that vascular structural changes may manifest as a result of hypertension during pregnancy and may be an adaptive response to increased arterial stress.

In the post-partum period, several studies report discrepant findings with respect to the persistence of increased CIMT following a pregnancy complicated by gestational hypertension or PE. A study of 22 early-onset PE cases found that CIMT was significantly increased in women at least 3 months postpartum by 73 μm (*p* < 0.001) as compared to 22 nulliparous women, but it was not found to be significantly increased compared to 22 women with a normotensive pregnancy (14). These findings suggest that an increased CIMT may reflect a normal physiological response to pregnancy rather than a pathological response to a hypertensive disorder of pregnancy. This is supported by an epidemiological study linking the number of births to a concurrent increase in CIMT in females over a 6 years follow-up period, suggesting that childbearing may independently impact the risk of cardiovascular disease (17). A more recent study of 34 women with previous PE, however, reported increased CIMT at least 12–24 months after pre-eclamptic pregnancy, suggesting that there is persistent structural arterial damage (18). Conversely, Yuan et al. refuted this finding, suggesting that the increase in CIMT seen during PE resolved by 18 months postpartum in their cohort. They suggested that carotid arterial remodeling may occur during pregnancy, but reverses or attenuates in the postpartum period (15). Interestingly, a prospective case-control study implementing a 12-weeks aerobic exercise training program in 24 women 6–12 months postpartum PE showed a significant decrease in CIMT after completing the training program (530 ± 138 vs. 580 ± 92 μm; *P* < 0.01). This decrease was also evident in healthy controls, suggesting that both groups can significantly decrease their cardiovascular risk profile postpartum (19).

The evidence with respect to the longer postpartum period is minimal. One study of women with a history of PE reported a significantly increased intima-media thickness ratio at both 1 and 7 years postpartum suggesting that there are still signs of sub-clinical atherosclerosis (20). Providing some clarity was a recent meta-analysis including a total of 813 women with a history of PE ≥ 10 years postpartum. This reported greater CIMT in the PE group compared to controls [0.18 mm (95% CI, 0.05–0.30 mm; *P* = 0.004)] (21). Women with a history of PE who were on anti-hypertensives, but had no prior diagnosed cardiovascular event, still had a greater CIMT than women who had no history of PE, suggesting minimal CIMT recovery and chronic vascular changes after PE.

Taken together, these studies highlight the need for more robust prospective studies assessing CIMT throughout the course of a PE pregnancy and long-term postpartum to clearly elucidate the time course and persistence of subclinical vascular changes. From a clinical and practical perspective, the user-dependence and varied acquisition protocols in CIMT measurement and the lack of evidence that using CIMT to guide clinical decisions results in better outcomes, makes this a challenging clinical tool to implement broadly to evaluate the extent of vascular changes in women who have been affected by PE.

#### Cardiac Computed Tomography and Calcium Score

Coronary artery calcification (CAC) is an important marker of CVD. Coronary calcium scores are measured by Agatston units (AU) using computed tomography (CT). The St. Francis Heart study determined that for a coronary calcium score >100 AU, the relative risk for atherosclerotic CVD was 9.6 (6.1–13.9; 95% CI) (22). Additionally, calcification is believed to contribute to arterial stiffness, which further confers an increased risk of CVD (23). There is currently no evidence with respect to coronary calcium scores in women preceding or during a pre-eclamptic pregnancy. This is understandable, given the risks of exposing young women or a developing fetus to radiation.

Five studies were identified evaluating CT coronary angiogram (CTCA) or calcium score postpartum in PE. The data in the postpartum period suggests a strong association between PE and vascular structural changes. Coronary calcification has been evaluated in 4 studies using formal CTCA and in 1 study using CAC score alone. In a recent multicentre prospective cohort study, 164 women aged 45–55 years old with a history of PE 10–20 years prior demonstrated a higher risk of CAC >0 AU as compared to the cohort studies in the Multi-Ethnic Study of Atherosclerosis (RR 1.7; 95% CI, 0.4–19.3) (24, 25). A further study of a population of 151 women with a history of hypertension during pregnancy found that the odds of having a higher CAC was 1.52 (95% CI, 0.96–2.39) greater after adjustment for BMI, waist-hip ratio, and systolic/diastolic blood pressure; suggesting that hypertension during pregnancy independently increases the risk of CVD (26). Similar findings were demonstrated in another sample of 51 Caucasian women with a history of hypertension during pregnancy, of which 24 had an identified history of PE. Hypertension during pregnancy remained significantly associated with CAC even with additional adjustment for serum creatinine levels, urinary albumin creatinine ratio, menopause status, diabetes status and antihypertensive medication use (27). Furthermore, in a sample of 40 women >30 years after affected pregnancy, the frequency of a CAC >50 AU was greater in women with a history of PE (23 vs. 0%; *P* = 0.001). This prospective cohort study found that the odds of having a higher CAC in women with a history of PE was 3.2 (95% CI, 1.21–8.49) and 2.61 (95% CI, 0.95–7.14) times greater when adjusted for BMI and current hypertension, respectively (28). Lending further weight to this argument is a study of postmenopausal women 35 years following PE. In this cohort of 37 women, there was a significant increase in CAC scores ranging from 0 to 25 (*P* = 0.026) compared to those without a history of PE (29).

Whether PE inherently alters the vascular system of women or rather pregnancy unmasks subclinical CVD and potentiates a pathological increase in blood pressure cannot be elucidated from these above CAC and CTCA studies. These, however, are potential tools to assess the long-term extent of vascular damage associated with this disease, and to assist in risk stratifying women.

#### Retinal Microvasculature

Assessment of retinal vessel architecture using fundus photography is a novel potential cardiovascular risk marker. Increasing evidence suggests that retinal microvascular abnormalities are a reflection of hypertension and other vascular risk factors such as hyperglycaemia, central obesity and dyslipidaemia (30). Specifically, narrowing of retinal arteriolar vessels and widening of venular vessels has been shown to be associated with increased risk of hypertension (31).

In total, there were five studies identified that evaluated retinal microvasculature as a marker of deteriorating vascular structure in PE. These studies suggested persistent structural changes evident prior to disease onset, during PE and postpartum. Normally, a decrease in blood pressure during a normotensive pregnancy correlates with a significant increase in retinal arteriolar and venular caliber, before returning to normal after pregnancy (32). In PE, the assessment of retinal arterial vessels has been utilized to potentially assess a vascular response to systemic changes induced by the release of inflammatory markers into the maternal circulation. In a particular cohort of 92 women, a significant reduction in the caliber of arteriolar and venular vessels at 13 and 19 weeks gestation was found in the 9 women that subsequently developed PE, as measured by central retinal arteriolar equivalent (CRAE) and central retinal venular equivalent (CRVE), respectively (33). These findings were coupled with an absence in the normal physiological drop in blood pressure, suggesting an increase in peripheral vascular resistance occurring prior to clinical diagnosis of PE. Abnormalities of the retinal vasculature have been shown to reflect the existence of prior or current hypertension and have been associated with endothelial dysfunction and inflammation potentiated by cardiovascular risk factors (34). Therefore, given this is the first study to record a structural abnormality so early on in pregnancy, this correlation is suggestive of either pre-existing maternal factors that contribute to increased risk of developing PE, or the onset of dynamic vascular changes before the clinical presentation of PE. This is of potential great importance and relevance to first trimester PE screening algorithms and warrants further investigation.

Similar findings have been found both during PE and at 1 year postpartum (35). A study of the same population both during and 1 year postpartum found a significant decrease in central retinal artery equivalent diameter (CRAE) and central retinal vein equivalent diameter (CRVE) during PE followed by a small recovery postpartum, however the decrease persisted. Furthermore, 63 women with a history of PE who were followed up at 6 years postpartum recorded a decrease in retinal arterial caliber (137.8 ± 14.4 *vs*. 145.8 ±16.9 μ*m*; *P* < 0.001). These findings remained when adjusted for various cardiovascular risk factors, including mean arterial pressure.

As a non-invasive, simple and inexpensive modality for assessing vascular resistance, this is a tool that requires further investigation. It has great potential to assist in the identification of women at risk of developing PE as well as a potentially simple clinical tool in monitoring consequential vascular damage in women previously affected by PE.

### Vascular Function

#### Flow Mediated Dilatation and Peripheral Arterial Tonometry

Brachial flow-mediated dilatation (FMD) is an established non-invasive, ultrasound technique that allows for the investigation of endothelial function and cardiovascular risk. It measures endothelial dependent dilatation by recording the change in brachial arterial diameter mediated by nitric oxide release in response to an increase in shear stress (36). Normally, pregnancy is associated with an increase in FMD, which reflects improved endothelial function. A meta-analysis of 23 studies including 14,753 subjects found that brachial FMD is inversely associated with future CVD events (37). As such, it has become a potential modality for examining endothelial function in disease states throughout pregnancy and postpartum. Peripheral arterial tonometry (PAT) is based on similar physiological mechanisms, where the brachial artery is occluded to elicit transient ischemia peripherally and stimulate reactive vasodilatation, before hyperemia is induced once the cuff is deflated. The difference in PAT is that the endpoint that is measured is arterial pulse volume amplitude in the finger to thereby calculate a reactive hyperemia index (RHI) (38). A meta-analysis of 6 PAT studies and 1,602 subjects predicted a decrease in the relative risk of cardiovascular events for every 0.1 increase in the logarithmic value of RHI (RR 0.79; 95% CI, 0.71–0.87) (39).

Twenty-seven studies of FMD and seven of PAT were identified in this systematic review, with some predominant findings related to endothelial dysfunction in PE. Studies in high risk women who consequently develop PE have demonstrated that endothelial dysfunction precedes onset of the clinical disease. A prospective study of 15 high risk women who subsequently develop PE found significantly decreased FMD compared to those who remained normotensive prior to clinical disease onset, particularly between 24 and 28 weeks gestation (3.6 ± 2.38% vs. 8.42 ± 3.15%; *P* = 0.001) (40). Moreover, sensitivity of FMD as a predictor for high risk pregnant women in developing PE was 87.5% for early-onset and 95.5% for late-onset when a decrease of <2.5% on FMD was detected between 16 and 19 weeks and 24–27 weeks, suggesting that FMD can become a potential tool in predicting PE (6). In a recent cohort of 62 women who screened high risk for the development of PE, the 10 women who subsequently developed early-onset PE illustrated a significant difference in FMD measurements at 24–27 weeks gestation and at delivery as compared to uncomplicated pregnancies. There was no meaningful difference, however, at 16–19 weeks gestation, suggesting the absence of prior endothelial dysfunction (41). With respect to PAT as a measure of endothelial function, however, a study of 180 women in whom PAT was measured at 16 and 28 weeks gestation, of the 24 women who subsequently developed PE there was no significant difference in RHI between the cases and controls at either 16 or 28 weeks gestation (42).

Whilst there is strong evidence to suggest that endothelial function as measured by FMD is decreased during a pre-eclamptic pregnancy (16, 43–45), the timing and magnitude of this change is unclear. Supporting this, PAT measured in 105 PE cases with an average gestational age of 30 weeks illustrated a significantly reduced RHI compared to 110 normotensive controls at 1.70 (1.04–3.61) vs. 1.81 (1.18–4.62) (*P* = 0.0269), respectively (46). Interestingly, FMD has been demonstrated to be significantly reduced in pre-eclamptic women who present with bilateral uterine artery notches compared to those without (47). Bilateral uterine artery notching, in turn, is associated with placental ischemia, suggesting that there is increased high resistance in the uteroplacental circulation. This is shown to be linked with a more severe degree of endothelial dysfunction, but whether it is associated with early or late-onset PE has not been elucidated.

Evaluation of endothelial function via FMD in the postpartum setting reveals that the dysfunction seen in PE likely persists beyond the time of pregnancy. In the short term, studies have demonstrated that FMD is significantly lower in pre-eclamptic women between 3 and 6 months postpartum (48). At 3 months postpartum, FMD measurements were significantly reduced in 20 women (*p* < 0.001), who also exhibited impaired diastolic and systolic left ventricular function during pregnancy, which interestingly did not persist concurrently with endothelial dysfunction (45). This finding, however, was not universally reported, with one study of 30 women reporting no significant difference in FMD at 1 month after delivery in both mild and severe PE (16). The majority of studies, however, report reduced FMD postpartum. For example, a study of 20 women 2–3 years postpartum, reported a reduced FMD (10.7 ± 8.6% vs. 17.9 ± 7.9%; *P* = 0.04) and arterial distensibility in women following PE, which was found to be proportionate to decreased infant birthweight (49–51).

In consolidation, a recent meta-analysis examining FMD throughout different time courses of PE concluded that FMD was reduced both before the clinical onset and during PE. This lower FMD persisted 3 years postpartum after excluding studies that included women with chronic hypertension and a history of smoking (52).

Evidence regarding the long-term persistence of endothelial dysfunction following a pre-eclamptic pregnancy remains unclear. Studies have shown endothelial dysfunction to persist up to 5 years postpartum (53–55). However, PAT measured in 26 women with previously affected pregnancies 5–8 years prior showed no significant difference in endothelial function (*P* = 0.7). Interestingly, the 11 women in the PE group who delivered a small for gestational age infant had a significantly lower RHI compared to the controls (*P* = 0.005), suggesting other factors such as low birth weight and preterm birth playing a role in persistent endothelial dysfunction (56). Studies that assessed FMD <10 years following the index PE pregnancy, do not support the hypothesis of persistent endothelial dysfunction mediating CVD risk. In 39 women 9–11 years postpartum PE, no significant difference in mean FMD was observed compared to women with uncomplicated pregnancies (8.28 ± 3.68% vs. 8.21 ± 4.02%; *P* = 0.90) (57). Further supporting this finding, another population of women followed up at both 1 year and 11 years postpartum PE reported that FMD was significantly decreased at 1 year, but had normalized at 11 years, therefore further suggesting endothelial function recovery over time (58).

#### Pulse Wave Analysis and Pulse Wave Velocity

Arterial stiffness is a pathological process that develops secondary to changes within the arterial system such as degeneration of elastin and increases in collagen, leading to a thickening of the arterial wall. Pulse wave velocity (PWV) is considered to be the most accurate non-invasive modality in evaluating arterial stiffness. Pulse wave analysis (PWA) is another modality which measures arterial function by deriving variables from arterial waveforms using applanation tonometry. These waveforms are characterized by variables including Augmentation Index (AIx), which is a measure of the proportion of the central pulse pressure attributed to the reflected pulse wave (59). A recent meta-analysis of 14,673 Japanese participants showed that an increase in brachial-ankle (ba)PWV independently predicted an increased risk of developing CVD (60). Similarly, a recent study of the Framingham Heart Study cohort found that high carotid-femoral (cf)PWV, adjusted for age and sex, was associated with significantly increased risk of a cardiovascular event and this was further increased when coupled with central pulse pressure (HR 1.79; 95% CI, 1.30–2.46) (61). As such, PWV is considered the gold standard in measuring arterial stiffness and a potential tool in monitoring vascular function within women affected by PE (59).

The key consistent findings of the 14 studies identified was an increase in cfPWV and AIx prior to disease onset, during and up to 2–3 years postpartum. Although there is little evidence, cfPWV has been previously demonstrated to be increased in women at 23 weeks gestation who subsequently developed PE (62). Further to this, there was no significant difference between women with early-onset and those with late-onset as well as those with abnormal and normal doppler uterine artery examination. CfPWV has been reported to be a strong predictor for the development of PE in high risk women when measured between 22 and 26 weeks gestation, especially in early-onset PE (63). These findings suggest that arterial stiffness pre-dates the development of the clinical presentation. To consolidate this finding, in a cross sectional study of a rural South African population, 85 women with PE displayed significantly increased cfPWV and AIx (64).

With respect to the postpartum period, Robb et al. reported that arterial stiffness increases in both normal pregnancy and PE compared to nulliparous women, however augmentation index and cfPWV had a significantly greater and more prolonged increase within the PE group, persisting up to 7 weeks postpartum (65). Similarly, a significantly increased AIx was reported in 20 women with previous PE 2–3 years postpartum (37.7 ± 5.1% vs. 23.8 ± 4.4%; *P* < 0.001) (49). Conversely, 30 women 5–6 years after the index pre-eclamptic pregnancy exhibited no significant increase in pulse-wave reflections, thereby suggesting there is no permanent difference in arterial distensibility postpartum (66). This was supported by 2 other observational cohort studies where PWV was measured at <10 years postpartum. There was no significant difference in cfPWV in the pre-eclamptic women analyzed in either of these studies (58, 67). Although a non-significant increase in PWV was reported, this may be correlated to an increase in blood pressure prevalent within the PE group (67).

**Figure 2:**
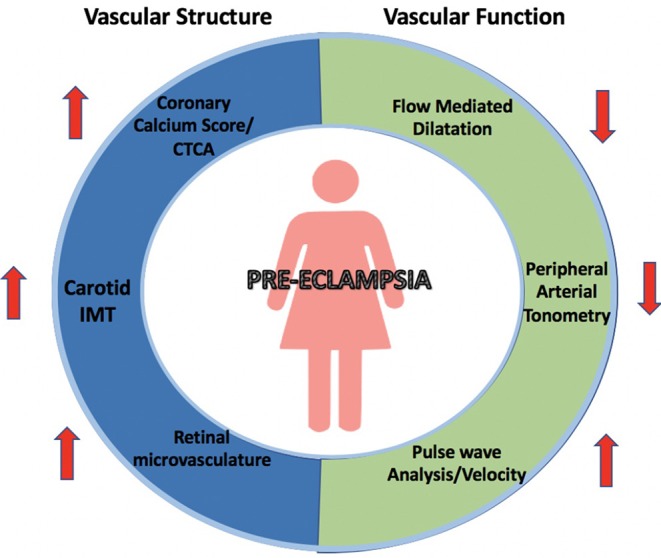
Changes in vascular structure and function associated with pre-eclampsia. Summary of measurements of vascular structural and functional changes illustrated by non-invasive modalities including coronary calcium scores and CT coronary angiograms, carotid IMT, retinal microvasculature, flow-mediated dilation, peripheral arterial tonometry and pulse wave velocity and analysis.

The key modalities used for non-invasively assessing vascular structure and function associated with PE are outlined in Figure 2. An overview of the available studies identified in the systematic review and samples sizes of each study are outlined in Table 2.

**Table 2:** Overview of studies evaluating vascular structure and function prior to, during and after pre-eclampsia.

**MODALITY**	**TIMEFRAME**		
	**Prior to PE**	**During PE**	**Postpartum**
Carotid Intima-Media Thickness	Brueckmann et al. (11) (143 PE cases)	Yuan et al. (15) (22 late-onset PE cases) Stergiotou et al. (13) (100 PE cases; 50 early onset and 50 late-onset) Blaauw et al. (14) (22 early-onset PE cases) Memari et al. (12) (21 PE cases) Mori et al. (16) (30 PE cases; 14 mild and 16 severe PE)	Akhter et al. (20) (23 PE cases) Aykas et al. (53) (25 PE cases) Christensen (67) (21 PE cases; 4 early-onset and 17 late-onset) Ciftci et al. (68) (33 mild PE) Garovic et al. (21) (40 PE cases; 813 PE cases in meta-analysis) Goynumer et al. (18) (34 PE cases) Sandvik et al. (57) (39 PE cases)
Coronary CT and coronary calcium score	No current data	No current data	Beckman et al. (29) (37 PE cases Cassidy-Bushrow et al. (27) (24 PE cases) Sabour et al. (26) (151 had a history of hypertension during pregnancy) White et al. (28) (40 PE cases) Zoet et al. (24) (164 PE cases)
Retinal microvasculature	Lupton et al. (33) (9 PE cases) Porto et al. (41) (10 early-onset PE)	Soma-Pillay et al. (35) (40 PE cases; 24 early-onset and 16 late-onset)	Benschop et al. (5) (63 PE cases) Soma-Pillay et al. (35) (40 PE cases; 24 early-onset and 16 late-onset)
Brachial flow mediated dilatation	Alves et al. (69) (9 early-onset and 22 late-onset PE) Brandao et al. (40) (15 cases; 6 early-onset and 9 late-onset) Brandao et al. (6) (19 PE cases; 8 early-onset and 11 late-onset) Mori et al. (16) (30 PE cases; 14 mild and 16 severe PE) Porto et al. (41) (10 early-onset PE) Savvidou et al. (70) (10 PE cases) Weissgerber et al. (52) (156 PE cases) Magee et al. (71) (6 PE cases)	Adali et al. (72) (35 PE cases) Brodszki et al. (47) (28 PE cases; 15 with bilateral uterine artery notches and 13 without) Guimaraes et al. (44) (42 PE cases) Hamad et al. (48) (35 PE cases; 8 early-onset and 27 late-onset) Mannaerts et al. (73) (33 PE cases) Mori et al. (16) (30 PE cases; 14 mild and 16 severe PE) Oliveira et al. (43) (40 PE cases) Tyldum et al. (45) (20 PE cases; 7 early-onset and 13 late-onset Weissgerber et al. (52) (333 PE cases)	Agatisa et al. (50) (16 PE cases) Aykas et al. (53) (25 PE cases) Barry et al. (74) (49 PE cases) Breetveld et al. (55) (67 PE cases) Goynumer et al. (18) (34 severe PE cases) Hamad et al. (48) (35 PE cases; 8 early-onset and 27 late-onset) Hamad et al. (51) (18 PE cases) Lopes van Balen et al. (54) (79 PE cases) Mori et al. (16) (30 PE cases; 14 mild and 16 severe PE) Ostlund et al. (58) (15 PE cases) Paez et al. (49) (20 PE cases) Sandvik et al. (57) (39 PE cases) Tripathy et al. (75) (45 PE cases) Tyldum et al. (45) (20 PE cases; 7 early-onset and 13 late-onset Weissgerber et al. (52) (429 PE cases <3 years postpartum; 325 PE cases >3 years postpartum) Yinon et al. (76) (24 PE cases; 15 early-onset and 9 late-onset)
Pulse wave analysis and pulse wave velocity	Katsipi et. al (63) (11 early-onset; 10 late-onset—cfPWV measured) Savvidou et al. (62) (29 PE cases—cfPWV measured) Magee et al. (71) (6 PE cases; cfPWV)	Franz et al. (77) (21 PE cases; 11 early-onset and 10 late-onset—AIx measured) Oylumlu et al. (78) (45 PE cases; cfPWV measured) Namugowa et al. (64) (85 PE cases; 64 early-onset and 21 late-onset—cfPWV measured) Robb et al. (65) (15 PE cases; 7 preterm and 8 term—cfPWV measured) Rönnback et al. (79) (26 PE cases—cfPWV measured)	Christensen (67) (21 PE cases; 4 early-onset and 17 late-onset—aortic PWV measured) Lampinen et al. (66) (30 PE cases—PWA assessed) Orabona et al. (80) (30 early-onset and 30 late-onset PE cases—cfPWV measured) Ostlund et al. (58) (15 PE cases—cfPWV measured) Paez et al. (59) (20 PE cases—cfPWV measured) Souwer et al. (81) (14 early-onset PE—PWA assessed)
Peripheral arterial tonometry	Carty et al. (42) (143 PE cases)	Kumer et al. (82) (26 PE cases) Mannaerts et al. (83) (14 PE cases) Mannaerts et al. (73) (33 PE cases) Meeme et al. (46) (105 PE cases)	Kvehaugen et al. (56) (26 PE cases) Orabona et al. (80) (60 PE cases; 30 early-onset and 30 late-onset)

#### Postulated Mechanisms Linking PE to Abnormal Vascular Structure and Function

During normal pregnancy, adaptations such as increased intravascular volume and a reduction in vascular resistance within the maternal circulation result in an overall decreased blood pressure. In PE, impaired placentation occurs as a result of a complex interplay between vascular, immunological, and genetic factors. This leads to the placenta releasing soluble, toxic, antiangiogenic factors in response to hypoperfusion leading to inflammation and maternal systemic disease (3, 84).

Soluble Flt1 is believed to be the predominant factor released in the pathogenesis of PE. It primarily counteracts the function of pro-angiogenic proteins that usually reduce microvascular resistance, including vascular endothelial growth factor (VEGF) and placental growth factor (PlGF), by binding to them and preventing their ability to act on the endothelium (3). The vascular effects of sFlt1 have been clearly elucidated in a mouse model where virally induced overexpression of sFlt-1 led to the development of hypertension and proteinuria (85). Soluble endoglin (sEng), a placenta-derived soluble TGF-β1(transforming growth factor-β1) inhibitor, is another anti-angiogenic factor involved in PE (3). It inhibits the TGF-β1 signaling pathway thereby preventing capillary tube formation and increasing vascular permeability. Its effect in pregnant rats has been shown to enhance the vascular impact of sFlt1, leading to severe PE (86).

An imbalance in these anti-angiogenic factors is seen in women who develop PE; prior to the clinical condition, during PE and postpartum. For example, in a cohort of 159 women, sFlt1 and sEng measured at 10–17 weeks gestation were significantly increased in the 21 women who subsequently developed PE as compared to those with a normal pregnancy outcome. In conjunction, the increase in sFlt1 and sEng was consistent with a significant decrease in FMD indicative of endothelial dysfunction (87). Interestingly, it was found that the rise in sFlt1 did not correlate with an increase in mean arterial pressure until the 26–33-weeks time point, suggesting that the increased blood pressure is a consequence of endothelial dysfunction. A meta-analysis investigating the predictive value of sFlt1/PlGF ratio in PE found a pooled sensitivity of 80% (95% CI, 68–88%) and a pooled specificity of 92% (95% CI, 87–96%), suggesting this ratio may be a useful tool during the clinical assessment of women during early pregnancy (88). Moreover, increases in sFlt1 are found to be greater in early-onset vs. late-onset PE (89). This is further highlighted in a study suggesting sFlt1 levels directly correlate with the severity of disease (90). More recently, a prospective cohort study of 46 women with suspected or confirmed PE found that sFlt-1/PlGF ratio >38 at 30 weeks gestation continues to double in subsequent weeks, possibly reflecting an amplification of the disease process, before rapidly decreasing postpartum (91).

There has been one report demonstrating that women 5–8 years postpartum PE have a significantly increased level of circulating sFlt1 (79.7 ± 15 vs. 70.9 ± 11.2 pg/mL; *P* = 0.02) (56), illustrating that women with a history of PE have a persistent antiangiogenic profile. The role of persistent circulation of antiangiogenic factors in PE and increased risk of CVD, however, remains unclear. Increase in circulating sFlt1 has been shown to be associated in the development of heart failure (92) and in patients immediately after myocardial infarction (93), suggesting sFlt1 may be released in response to hypoperfusion or pain. However, the mechanism for sFlt1 release and endothelial interactions remain incompletely understood. In light of the findings noted above, sFlt1 may be associated with an overall acceleration in endothelial dysfunction and enhanced adverse vascular outcomes.

Likewise, expression of endoglin within the circulation is thought to be in response to endothelial damage and inflammation (94). Increased levels of soluble endoglin have been correlated with hypertension and diabetes, and to have a positive association with increased PWV and retinopathy, suggesting that endoglin plays a vital role in vascular function and development of disease (95). Similarly, elevation of sEng levels has been shown to be an indicator for major adverse cardiovascular events in patients with chronic coronary artery disease, suggesting increased levels may correlate to greater vascular damage, thereby resulting in an increased risk of vascular failure (96).

#### Clinical Considerations

The ability to identify women at risk of PE prior to disease onset would be extremely valuable but remains elusive, particularly for late onset PE. First trimester screening using multi-variable risk prediction models based primarily on placental factors has proven partially successful in identifying women at risk of early onset PE but has a low predictive value in late onset disease (97). A proposed reason for this is that these algorithms fail to appropriately account for maternal endothelial function, with the only included maternal vascular marker a peripheral blood pressure measurement. Non-invasive vascular assessment using one of the above methodologies may have a role to play in this risk prediction model. Given the technical expertise, time and user dependence of FMD and PWV these may not be the most user friendly and practical options. Retinal photography, however, is relatively inexpensive, fast and reproducible and thus has the potential to be of benefit, in conjunction with current risk prediction models, to help predict risk of PE in women. This hypothesis, however, requires testing and confirmation.

In the long-term following PE there is no clear clinical pathway for maternal cardiovascular follow-up. Beyond intensive primary prevention and close control of modifiable risk factors such as fasting cholesterol, glucose and bodyweight; CTCA and CAC may play an important role. A careful balance, however, must be struck between the predictive benefit and the risks of overdiagnosis and exposing relatively young women to ionizing radiation.

## Conclusions

PE is associated with an increased risk of premature CVD in woman, independent of concomitant risk factors. Studies suggest that this condition is associated with subclinical changes in vascular structure, such as an increase in CIMT and retinal microvascular caliber during pregnancy, and long-term elevations in coronary calcification. Abnormal endothelial function has also been demonstrated through reductions in flow mediated dilatation and increased PWV and AIx, however the timing and persistence of these changes is unclear. The pathophysiology linking PE with CVD is yet to be fully elucidated but has been postulated to involve inflammation and endothelial dysfunction. Whether PE initiates these pathologic changes or acts as a stress test unmaking latent disease in at-risk women is yet to be determined.

## Author Contributions

SK was responsible for the systematic review, synthesis of information, and drafting of the manuscript. MS and SP were responsible for review analysis, synthesis, and manuscript preparation. CA was responsible for the concept design, synthesis, analysis, and drafting of the manuscript. All authors approve the paper for submission.

### Conflict of Interest

The authors declare that the research was conducted in the absence of any commercial or financial relationships that could be construed as a potential conflict of interest.
